# CXCL13 Neutralization Attenuates Neuropsychiatric Manifestations in Lupus-Prone Mice

**DOI:** 10.3389/fimmu.2021.763065

**Published:** 2021-11-12

**Authors:** Michelle W. Huang, Ariel D. Stock, Chaim Putterman

**Affiliations:** ^1^ Department of Microbiology and Immunology, Albert Einstein College of Medicine, Bronx, NY, United States; ^2^ Department of Neurological Surgery, Montefiore Medical Center, Bronx, NY, United States; ^3^ Division of Rheumatology, Albert Einstein College of Medicine, Bronx, NY, United States; ^4^ Azrieli Faculty of Medicine, Bar-Ilan University, Zefat, Israel; ^5^ Galilee Medical Center Research Institute, Nahariya, Israel

**Keywords:** CXCL13, SLE, neuropsychiatric lupus (NPSLE), MRL/lpr, tertiary lymphoid structures

## Abstract

Neuropsychiatric lupus (NPSLE), the nervous system presentation of systemic lupus erythematosus (SLE), remains challenging to treat due to its unclear pathogenesis and lack of available targeted therapies. A potential contributor to disease progression is brain tertiary lymphoid structures (TLS); these ectopic lymphoid follicles that can develop tissue-targeted antibodies have recently been described in the MRL/*lpr* lupus mouse strain, a classic model for studying NPSLE. The brains of MRL/*lpr* mice show a significant increase of CXCL13, an important chemokine in lymphoid follicle formation and retention that may also play a role in the disease progression of NPSLE. The aim of the present study was to inhibit CXCL13 and examine the effect of this intervention on lymphoid formation and the development of neurobehavioral manifestations in lupus mice. Female MRL/*lpr* mice were injected with an anti-CXCL13 antibody, an IgG1 isotype-matched antibody, or PBS either three times a week for 12 weeks intraperitoneally (IP) starting at 6-8 weeks of age, or continuously intracerebroventricularly (ICV) with an osmotic pump over a two-week period starting at 15 weeks of age. Cognitive dysfunction and depression-like behavior were assessed at the end of treatment. When treatment was delivered IP, anti-CXCL13 treated mice showed significant improvement in cognitive function when compared to control treated mice. Depression-like behavior was attenuated as well. Furthermore, mice that received anti-CXCL13 by the ICV route showed similar beneficial effects. However, the extent of lymphocyte infiltration into the brain and the general composition of the aggregates were not substantively changed by anti-CXCL13 irrespective of the mode of administration. Nevertheless, analysis of brain gene expression in anti-CXCL13 treated mice showed significant differences in key immunological and neuro-inflammatory pathways that most likely explained the improvement in the behavioral phenotype. Our results indicate that CXCL13 affects the behavioral manifestations in the MRL/*lpr* strain and is important to the pathogenesis of murine NPSLE, suggesting it as a potential therapeutic target.

## 1 Introduction

Systemic lupus erythematosus (SLE) is an autoimmune disease involving multiple tissues and organ systems, including kidneys, joints, skin, blood cells, and brain (1). Mechanisms of disease, however, can vary depending on the system affected. Manifestations of SLE in the nervous system are termed neuropsychiatric lupus (NPSLE), which can affect roughly 20% to 40% of patients (2). Common presentations of NSPLE include depression, cognitive dysfunction, and anxiety (3, 4), but unfortunately, most treatments in current use are non-specific for the neuropsychiatric disease itself or only treat the symptoms and not the underlying disease. One major difficulty with developing an effective and targeted treatment is that the pathogenesis of NPSLE has yet to be fully elucidated.

The blood-brain barrier has traditionally been believed to be the site of entry for immune cells and inflammatory mediators that initiate NPSLE (5). Nevertheless, leukocyte accumulation and immune complex deposition at another key brain barrier, the blood-cerebrospinal fluid barrier (i.e. the choroid plexus), has been described in human NPSLE (6, 7), suggesting possible involvement of lymphocyte aggregation at the choroid plexus in the pathogenesis of lupus-associated brain disease. Moreover, we found in lupus mice that choroid plexus lymphocytes organize into tertiary lymphoid structures (TLS), lymphoid aggregates that develop in response to chronic, unresolved inflammation (8).

TLS functionally and structurally resemble secondary lymphoid organs but differ in that they form after development in non-lymphoid tissues and resolve with the inflammation (9). Though the initial events involved in TLS formation are still being investigated, it is clear that chemokines such as CXCL13 are overexpressed to recruit and retain immune cells after initiation (10). TLS then organize into B and T cell zones and can eventually develop germinal centers, where they can produce autoantibodies specific for the local tissue that can worsen disease and perpetuate inflammation (11). Additionally, the complexity and size of the TLS affect the local production of homeostatic cytokines and chemokines (such as CXCL13), which can continue to damage surrounding tissue (9).

Chemokine CXC ligand 13 (CXCL13; also, B lymphocyte chemoattractant (BLC)) is a chemokine important in lymphocyte recruitment and retention and lymphoid formation (12). This key chemokine is primarily produced by stromal cells and follicular dendritic cells and acts to recruit B cells and T follicular helper (Tfh) cells *via* its receptor, CXCR5. Indeed, the lack of CXCL13 or CXCR5 alone is enough to disrupt normal lymph node formation, while overexpressing CXCL13 is sufficient to induce lymphoid formation (13, 14). CXCL13 has also been shown to be important in SLE; systemic levels of CXCL13 positively correlate with disease activity scores in SLE patients, and blocking CXCL13 in lupus mice attenuated renal disease (15–17). These findings strongly suggest CXCL13’s involvement in both TLS formation and disease progression, but its role has not been studied in the context of neuropsychiatric involvement.

We used a spontaneous SLE mouse model, MRL/MpJ-*Fas^lpr^* (MRL/*lpr*), which presents with prominent neuropsychiatric manifestations. This strain has a mutation of the *FAS* gene that disrupts normal Fas-mediated apoptosis and results in ineffective clearing of autoreactive lymphocytes, leading to nuclear autoantibody production, hypergammaglobulinemia, lymphadenopathy, skin, and kidney disease (18). MRL/*lpr* mice also exhibit neurobehavioral abnormalities reminiscent of human NPSLE, including depression-like behavior and cognitive abnormalities in the form of memory impairment (18, 19). In this study, we neutralized CXCL13 to block lymphocyte recruitment and organization to understand the contribution of this process to the neuropsychiatric manifestations seen in MRL/*lpr* mice. MRL/*lpr* were treated with an anti-CXCL13 antibody delivered systemically or locally (in the brain), and assessed for changes in the behavioral phenotype to determine the potential of blocking CXCL13 as a therapeutic modality for NPSLE.

## 2 Methods and Materials

### 2.1 Mice

Female MRL/*lpr* mice were purchased from Jackson Laboratory (Bar Harbor, ME) and housed at the Albert Einstein College of Medicine animal facility (Bronx, NY). This study utilized two different delivery methods of the treatment: intraperitoneally (IP) or intracerebroventricularly (ICV). Both delivery methods were tested using three cohorts of mice each (n=18-21 per cohort; n=4-7 mice per group per cohort) that were run independently to establish the reproducibility of our findings. Cohorts of the same delivery method were combined after establishing no significant differences between cohorts. All animal protocols were approved by the Institutional Animal Care and Use Committee at Albert Einstein College of Medicine.

For the IP cohort, mice were injected intraperitoneally starting at 6-8 weeks of age with 100 µg of an anti-CXCL13 antibody (IgG1; Vaccinex, Inc., Rochester, NY), 100 µg of the IgG isotype control antibody (Vaccinex), or sterile PBS, three times a week for 12 weeks. The PBS control group served as a baseline for comparison. The antibody dose was determined based on its measured half-life in mice (data not shown) and a previous study showing attenuation of disease in an autoimmune murine model using this dosage and frequency (20).

For the ICV cohort, the mice underwent surgery at 15 weeks of age to implant a single cannula into the right ventricle by the following coordinates (derived from Paxinos and Franklin’s the Mouse Brain in Stereotaxic Coordinates, Compact, 3^rd^ edition): anteroposterior: -0.34 mm, mediolateral: 1.0 mm, and dorsal ventricular: 2 mm. Previous studies in the MRL/*lpr* strain using these coordinates confirmed correct localization into the ventricles (21). The cannulas (Brain Infusion Kit 3; Alzet, Cupertino, CA) were connected to a mini-osmotic pump (Model 2002, Alzet, Cupertino, CA) that delivered a volume of 0.5 µl/hour, such that the treatment groups received a total of 60 µg/week of either anti-CXCL13 antibody or the isotype control. The particular dose chosen for the ICV treatment regimen was selected based on average serum concentrations achieved following intraperitoneal antibody injection (22). This rodent study found that about 16-20% of the total antibody dose delivered intraperitoneally was detected in the serum over time; the amount of antibody delivered ICV was therefore set at 20% of the weekly IP dosage. A subset of the mice that did not receive any surgery (“non-surgery” group) served as a group for baseline comparison.

When only a subset of mice in any given group or cohort was used in a specific experiment, mice were selected randomly for inclusion.

### 2.2 Total IgG and Anti-Double-Stranded DNA (Anti-dsDNA) Serum Titers

Serum levels of total IgG and anti-dsDNA antibodies were measured by enzyme-linked immunosorbent assay (ELISA), as previously described (23, 24).

### 2.3 Behavioral Testing

All mice were tested for cognitive function, using the object placement (OP) and object recognition (OR) tasks, and depression-like behavior, using the Porsolt swim test. All tests were done at 17 weeks (ICV cohort) or 18-20 weeks (IP cohort) of age. Mice were placed in the testing room at least 30 minutes before the start of each test to acclimate.

#### 2.3.1 Object Placement (OP) and Object Recognition (OR) Tasks

Both tasks required two trials: a training trial and a testing trial. For the training trial, the mice were exposed to and allowed to explore two identical objects in an arena before being removed. After a set retention interval, the mice were placed back into the arena for the testing trial, except one of the objects has either been moved (OP) or replaced with a similar, but not identical, object (OR). The object that had been placed in the new location (in the OP test) or was switched (in the OR test) compared to the training trial was deemed the “novel” object in the testing trial.

For the OP task, mice were trained for 5 minutes, retained for 20 minutes, and then tested for 4 minutes. For the OR task, mice were trained and tested for 4 minutes each and retained for 60 minutes in-between the two trials. These times were consistent among cohorts.

Exploration was assessed by manually timing physical contact with the object. Preference scores (%) were calculated as the percentage of time spent with the novel placement (OP) or object (OR) over total time spent with both objects. Scores ≥55% were considered as showing a passing preference, which was previously determined as a robust cutoff for these tasks in this strain (21).

#### 2.3.2 Porsolt Swim Test

Mice were individually tested by being placed in a clear tank containing water at 27°C for a total of 10 minutes. The first minute was not scored to allow adjustment; the next 9 minutes were scored manually in 3-minute time bins. Each test was also recorded by video with the Viewer III software. Percent immobility was calculated as a percentage of time spent immobile over total time scored.

### 2.4 Renal Function Assessment

Blood urea nitrogen (BUN) levels were measured using the DIUR 500 kit from BioAssay Systems (Hayward, CA), per the manufacturer’s instructions. Proteinuria levels were semi-quantitatively determined using Uristix test strips (Siemens Healthcare Diagnostics, Tarrytown, NY).

### 2.5 Histology and Immunofluorescence Staining

#### 2.5.1 Slide Preparation and Hematoxylin and Eosin (H&E) Staining

Paraffin embedding, sectioning, and staining with H&E were done by the Histopathology and Comparative Pathology Core at the Albert Einstein College of Medicine.

#### 2.5.2 Histology Scoring

H&E sections were blindly scored semi-quantitatively by the Histopathology and Comparative Pathology Core at the Albert Einstein College of Medicine, focusing on cellular infiltration and resulting stromal expansion. Scores were given using the following gradation: 0 = normal, 1 = minimal, 2 = mild, 3 = moderate, 4 = marked/severe.

#### 2.5.3 Immunofluorescence Staining

Brain tissue fixed in paraformaldehyde was embedded in paraffin and coronally sectioned for immunofluorescent staining. All sections were deparaffinized and rehydrated, heated with either citrate buffer (pH 6) or Tris-EDTA buffer (pH 9) for antigen retrieval, and then blocked with 20% normal horse serum. Depending on the stain, sections were incubated with primary antibodies of:

(1) rat anti-mouse B220 (1:100; BD, Franklin Lakes, NJ) and rabbit anti-mouse CD3e (1:100; Invitrogen, Waltham, MA), followed by the secondary antibodies Alexa Fluor 488-conjugated donkey anti-rat and Cy5-conjugated donkey anti-rabbit (both 1:100), respectively;

(2) rabbit anti-mouse syndecan-1 (SDC1) (1:100; Sino Biological, Wayne, PA) and Alexa Fluor 594-conjugated donkey anti-mouse IgG (1:300), followed by the secondary antibodies Alexa Fluor 488-conjugated donkey anti-rabbit (1:100) and Alexa Fluor 594-conjugated donkey anti-mouse IgG (1:500), respectively;

(3) rat anti-human/mouse activation-induced cytidine deaminase (AID) (1:50; Invitrogen, Waltham, MA), followed by biotin-conjugated donkey anti-rat and amplified by the Tyramine Signal Amplification (TSA) Cyanine 5 System from Perkin Elmer (Waltham, MA).

All fluorophore-conjugated antibodies were from Jackson ImmunoResearch (West Grove, PA).

#### 2.5.4 TUNEL Staining

TUNEL (terminal deoxynucleotidyl transferase dUTP nick end labeling) immunohistochemical staining was done by the Laboratory of Comparative Pathology at Memorial Sloan Kettering Cancer Center, New York. Cells were counted using the QuPath program, version 0.2.3.

#### 2.5.5 Slide Imaging and Analysis

Stained slides were imaged using the Invitrogen EVOS FL Auto 2 Cell Imaging System and then analyzed using ImageJ (National Institutes of Health, Bethesda, MD).

### 2.6 RNA Isolation and Polymerase-Chain Reaction (PCR) Array

A subset of each cohort chosen randomly had brain tissue taken for RNA processing. All RNA was extracted with TRIzol reagent and isolated using the Zymo Research Direct-zol RNA Miniprep Plus Kit (Irvine, CA). cDNA was prepared using the Qiagen RT^2^ First Strand Kit and then processed through the Qiagen RT^2^ Profiler PCR Array, Mouse Multiple Sclerosis [PAMM-125Z] (Hilden, Germany), as specified by the manufacturer’s instructions. The data was analyzed using manufacturer’s provided online tool. Enrichment analyses were done by using Enrichr and sorted by Biological Processes (2021 version) (25–27).

### 2.7 Statistics

GraphPad Prism 9 software (La Jolla, CA) and JMP 15 (SAS, Cary, NC) were used to analyze the data. Normally distributed data were compared by ANOVA with Tukey’s multiple comparisons test; non-normally distributed data were compared by ANOVA with Dunn’s multiple comparisons test. Significance was considered as p<0.05. OP and OR tasks were analyzed using the Pearson chi-square test. All graphs are shown as mean ± SEM.

## 3 Results

### 3.1 Blocking CXCL13 Systemically Did Not Change Systemic Disease Development

**Figure 1:**
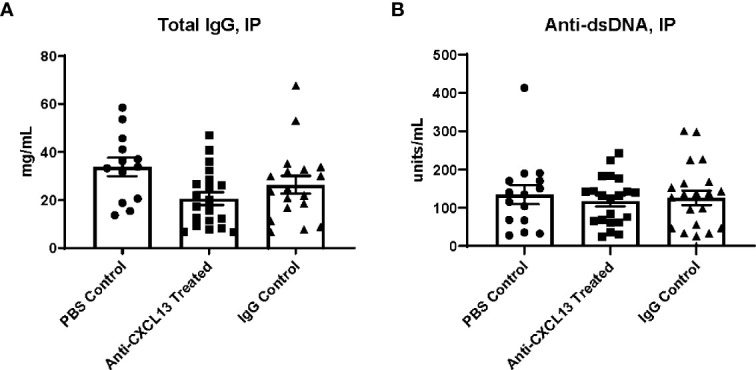
Terminal serum antibody levels. **(A)** Total IgG and **(B)** anti-dsDNA antibody levels were determined by ELISA using terminal serum of the mice. A positive control was used to normalize the anti-dsDNA antibody levels. Comparisons between the anti-CXCL13 treated group and IgG control group were not significantly different (Total IgG: Anti-CXCL13 Treated: 20.64 ± 2.67 mg/mL and IgG Control: 26.39 ± 3.68 mg/mL, p=0.41; Anti-dsDNA: Anti-CXCL13 Treated: 117.70 ± 13.74 units/mL and IgG Control: 126.10 ± 18.69 units/mL, p>0.99). Total IgG: PBS control: n=13, Anti-CXCL13 treated: n=20, IgG control: n=18. anti-dsDNA: PBS control: n=15, Anti-CXCL13 treated: n=20, IgG Control: n=21. Anti-dsDNA, anti-double stranded DNA.

Female MRL/*lpr* mice were treated with an anti-CXCL13 neutralizing antibody, an isotype-matched IgG control antibody, or sterile PBS three times a week intraperitoneally. Treatment started at 6-8 weeks of age and continued for 12 weeks, ending around 18-20 weeks of age. MRL/*lpr* mice display hypergammaglobulinemia and increased titers of anti-nuclear antibodies as indicators of systemic humoral autoimmunity (28, 29). Since in the cohort of mice treated intraperitoneally treatment was delivered systemically, inhibiting CXCL13-mediated pathways in this fashion was anticipated to disrupt systemic disease progression. We determined levels of antibodies and autoantibodies in terminal serum taken at 18-20 weeks of age for the IP cohort and found that total IgG levels were not significantly different between the anti-CXCL13 and isotype control treated groups (p=0.41) (**Figure 1A**). Anti-dsDNA antibody levels also did not differ between the groups (vs. IgG: p>0.99; **Figure 1B**). When individual IgG isotypes were measured, no significant differences were found as well (data not shown). Additionally, neither dermatitis nor lymphadenopathy was affected by treatment, as determined *via* skin macroscopic and histological appearance and lymph node weights, respectively (data not shown). Therefore, blocking CXCL13 did not significantly affect systemic disease in this mouse model.

### 3.2 Renal Function Was Unaltered With Treatment Delivered IP

**Figure 2:**
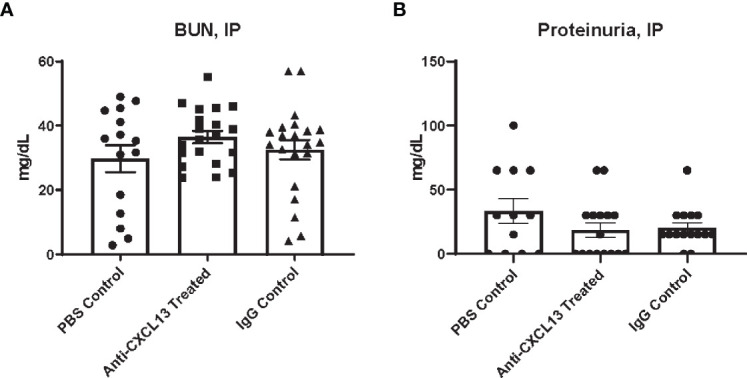
Renal function assessment. Renal function at sacrifice was determined by levels of **(A)** urea nitrogen in the blood and **(B)** protein in the urine. Neither measure showed a significance difference in renal function among groups (BUN: Anti-CXCL13 Treated: 36.48 ± 1.94 mg/dL and IgG Control: 32.53 ± 3.05 mg/dL, p>0.99; Proteinuria: Anti-CXCL13 Treated: 18.44 ± 5.68 mg/dL and IgG Control: 20.33 ± 4.04 mg/dL, p>0.99). BUN: PBS control: n=15, Anti-CXCL13 treated: n=20, IgG control: n=21. Proteinuria: PBS control: n=12, Anti-CXCL13 treated: n=16, IgG Control: n=15. Some samples were excluded because of the limited amount to run the analysis for proteinuria (3 samples from the PBS control, 4 from anti-CXCL13 treated, and 6 from IgG control). BUN, blood urea nitrogen.

Another disease progression indicator expected to be altered with systemic CXCL13 blockade is renal function. Renal inflammation [a.k.a. lupus nephritis (LN)] is a common manifestation in SLE patients and is also prominent in the MRL/*lpr* strain. Elevated levels of urea in the blood and protein in the urine are indicators of kidney dysfunction (30). Serum and urine were collected at the end of treatment, at 18-20 weeks of age, for analysis. When delivering treatment IP, blood urea nitrogen (BUN) levels were similar when comparing the anti-CXCL13 to the IgG control treated group (p>0.99; **Figure 2A**). Proteinuria similarly showed no significant differences (vs. IgG: p>0.99; **Figure 2B**). Both these measures suggest that there were no functional differences in the kidneys after blocking CXCL13.

### 3.3 Behavioral Deficits Improved With CXCL13 Neutralization Systemically

**Figure 3:**
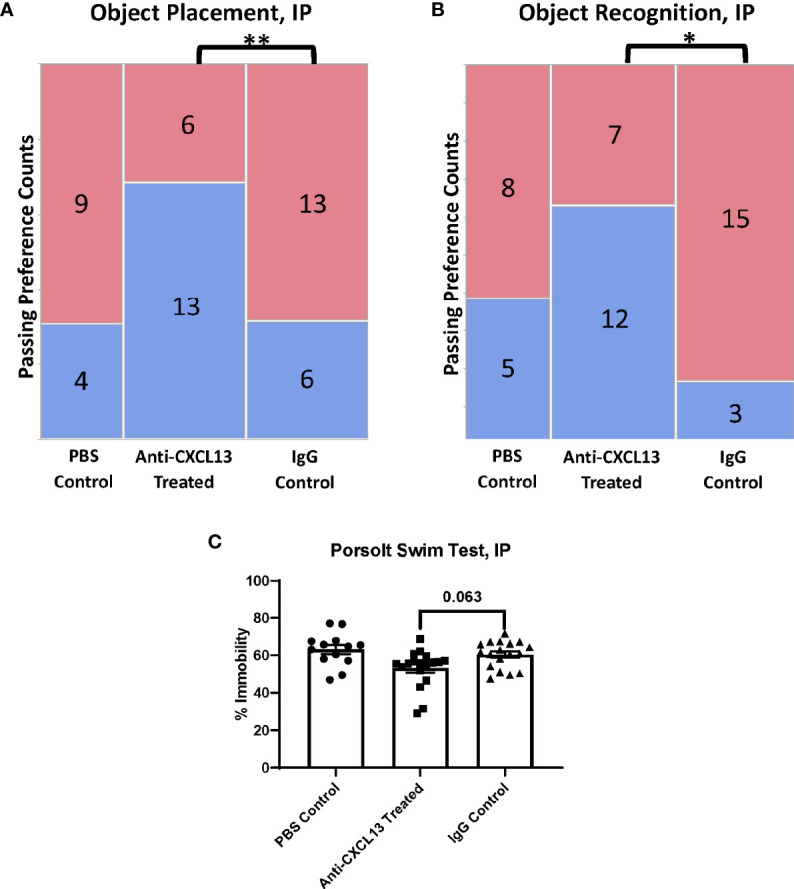
Assessment of behavioral manifestations. The object placement (OP) and object recognition (OR) tasks assess spatial and recognition memory impairment, respectively. **(A, B)** The anti-CXCL13 treated group had more mice show a passing preference (blue) for the novel placement or object compared to the IgG control group in both memory assessment tasks. OP task: **p = 0.0031; PBS control: n=13, Anti-CXCL13 treated: n=19, IgG control: n=19. OR task: *p = 0.022; PBS control: n=13, Anti-CXCL13 treated: n=19, IgG control: n=18. One mouse from the IgG control group was excluded from the OR results due to an inadequate exploration time during the training trial. **(C)** Depression-like behavior, determined *via* the Porsolt swim test, trended towards significance compared to the IgG control group. Anti-CXCL13 Treated: 53.07 ± 2.35% and IgG Control: 60.33 ± 1.68%; p=0.063. PBS control: n=13, Anti-CXCL13 treated: n=18, IgG control: n=18. *p < 0.05, **p ≤ 0.01.

The focus of our study was to determine how blocking CXCL13 affects neuropsychiatric manifestations. The MRL/*lpr* strain presents with cognitive dysfunction and depression-like behavior, manifestations that mirror common symptoms seen in NPSLE (19). The mice were tested during the last week of treatment at around 17-19 weeks of age. Treatment was given at the end of each day, after that day’s assessment, to ensure the injection process itself did not alter the behavioral results. The object placement (OP) and object recognition (OR) tasks assess spatial and recognition memory impairment, respectively. Memory is measured by preference for the novel placement or object, since, when given a choice, mice prefer to explore novel placements or objects over familiar ones. For the OP task, 13 of the 19 mice (68.4%) treated with anti-CXCL13 IP passed, as compared to only 6 of the 19 mice (31.6%) in the IgG control group (**p=0.0031) (**Figure 3A**; **Supplementary Figure 1A**). The performance in the OR task was also significantly improved in the treated mice when directly compared to the control group (vs. IgG: *p=0.022; **Figure 3B**; **Supplementary Figure 1A**). These results overall indicate that neutralizing CXCL13 improved both spatial and recognition memory compared to control treated mice.

The Porsolt swim test evaluates depression-like behavior in rodents; the MRL/*lpr* mice have been shown to demonstrate this manifestation as early as 5-6 weeks of age (19). This test is progressive, meaning the higher percentage of time spent immobile in the water indicates more severe behavioral despair. The anti-CXCL13 treated group trended towards significance compared to the IgG control treated group (p=0.063; **Figure 3C**). Thus, neutralizing CXCL13 may also have diminished depressive-like behavior in lupus mice.

### 3.4 Brain Infiltrate Extent and Composition

**Figure 4:**
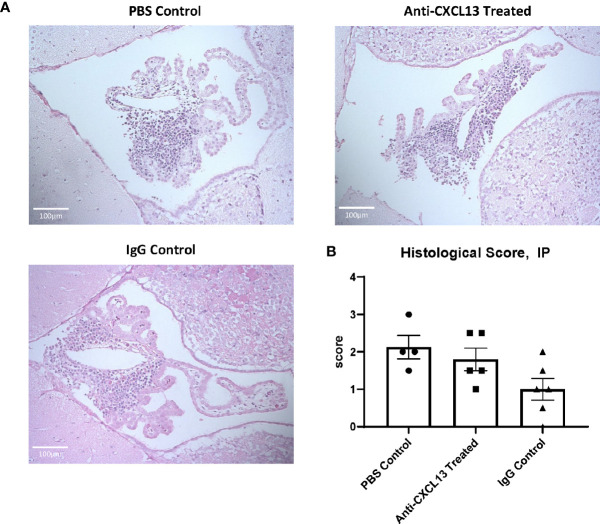
Choroid plexus infiltration. The extent of cellular infiltration in the choroid plexus was similar between anti-CXCL13 and IgG treated groups as evident by **(A)** hematoxylin and eosin (H&E) staining or **(B)** blind scoring by a veterinary pathologist (Anti-CXCL13 Treated: 1.80 ± 0.30 and IgG Control: 1.00 ± 0.29, p=0.17). Representative images of the third ventricle are shown from each group, taken at 20x magnification. Scores are taken from a representative group of the whole cohort. PBS control: n=4, Anti-CXCL13 treated: n=5, IgG control: n=6.

**Figure 5:**
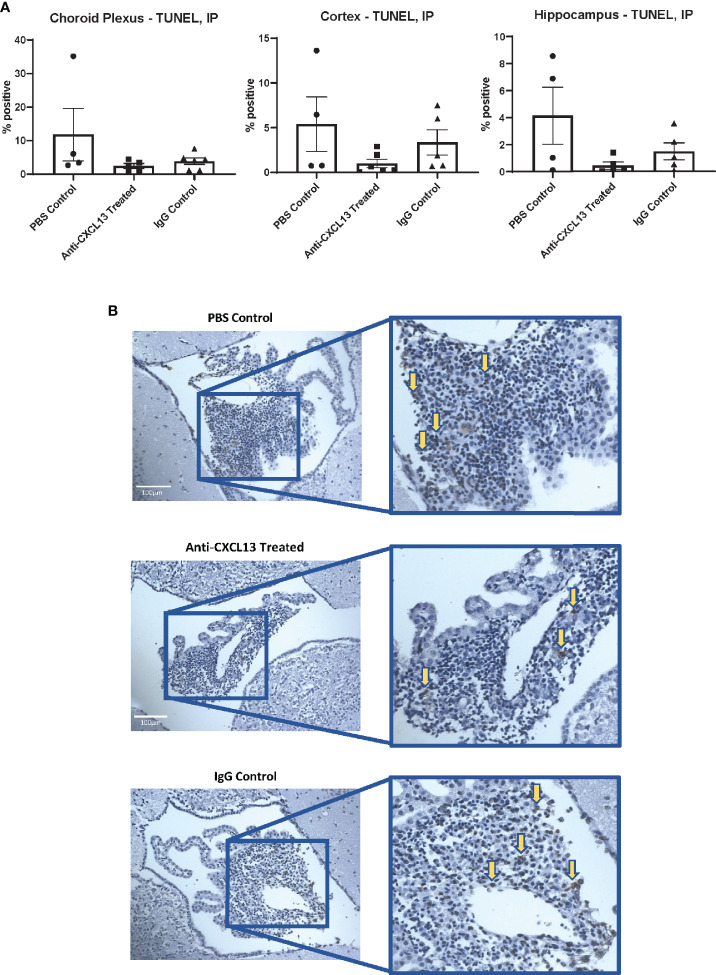
Apoptotic cells in the brain. **(A)** The percentages of apoptotic to total cells in different brain regions were not significantly different when comparing the groups to one another. The choroid plexus, cortex, and hippocampus were analyzed separately. (Choroid Plexus: Anti-CXCL13 Treated: 2.54 ± 0.73% and IgG Control: 3.87 ± 1.00%, p=0.77; Cortex: Anti-CXCL13 Treated: 1.00 ± 0.47% and IgG Control: 3.36 ± 1.41%, p=0.55; Hippocampus: Anti-CXCL13 Treated: 0.44± 0.26% and IgG Control: 1.50 ± 0.63%, p=0.77). **(B)** Representative images of the choroid plexus are shown, taken at 20x magnification. Arrows point to several representative positively stained cells for illustration. Percentages were determined by averaging 3-4 images each from a subset of representative mice from the cohort. PBS control: n=4, Anti-CXCL13 treated: n=5, IgG control: n=6. TUNEL, terminal deoxynucleotidyl transferase dUTP nick end labeling.

MRL/*lpr* mice show extensive immune cell infiltration in the choroid plexus (at the blood-cerebrospinal fluid barrier), organizing as TLS (8). Since TLS are damaging in autoimmune conditions, CXCL13 neutralization aimed to disrupt the recruitment and organization of these cells in the choroid plexus. Histological analysis showed extensive infiltration of immune cells in the choroid plexus in the PBS control group, as expected, but the extent of these aggregates was not different in any of the groups, as evident by representative images and their histology score (vs. IgG: p=0.17; **Figures 4A, B**). We also looked at potential differences in cell death. Since brain cellular apoptosis was not limited to the infiltration, the brain was divided into three regions – cortex, hippocampus, and choroid plexus – and TUNEL-positive cells were counted for each. The results are summarized in **Figure 5**. Focusing on the choroid plexus, the IP cohort saw no significant difference in the percentage of TUNEL-positive cells localized in the infiltrate (vs. IgG: p=0.77). The cortex and hippocampus also did not have differences in TUNEL-positive cells among the groups.

**Figure 6:**
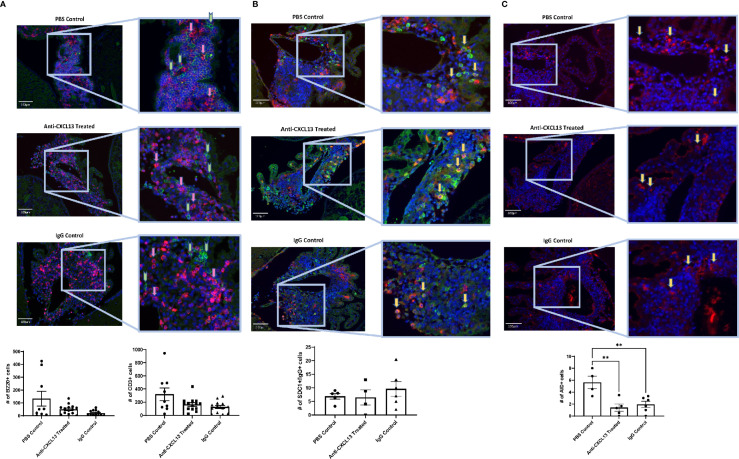
Choroid plexus infiltration composition. As with the extent of infiltration, the composition of the lymphocyte aggregates in the choroid plexus showed similar numbers of different cell types present, including **(A)** B cells [B220+(green); Anti-CXCL13 Treated: 50.23 ± 9.83 cells and IgG Control: 20.97 ± 6.07 cells, p=0.080] and T cells [CD3+(magenta); Anti-CXCL13 Treated: 166.00 ± 26.48 cells and IgG Control: 127.50 ± 24.89 cells, p>0.99], as well as **(B)** plasma cells [SDC1+(green)/IgG+(magenta); Anti-CXCL13 Treated: 6.44 ± 2.82 cells and IgG Control: 9.58 ± 2.69 cells, p=0.64]. **(C)** The PBS control group has more cells undergoing somatic hypermutation [AID+(magenta)] than either of the two antibody groups (PBS Control: 5.63 ± 1.05 cells, Anti-CXCL13 Treated: 1.40 ± 0.60 cells, and IgG Control: 1.94 ± 0.53 cells; PBS Control vs. Anti-CXCL13 Treated: **p=0.0041, PBS Control vs. IgG Control: **p=0.0082, Anti-CXCL13 Treated vs. IgG Control: p=0.83). Representative images are shown, taken at 20x magnification. Arrows point to several representative positively stained cells for illustration. The B220+ and CD3+ images have green and pink arrows to represent the two cell types, respectively. Cell counts are averages of 3-4 images per mouse, from a representative subset of mice from the cohort. B220/CD3: PBS control: n= 9, Anti-CXCL13: n=14, IgG control: n=13. SDC1/IgG & AID, PBS control: n=4-5, Anti-CXCL13 treated: n=4-5, IgG control: n=6. SDC1, syndecan-1; AID, activation-induced cytidine deaminase.

Since the extent of the infiltration and number of apoptotic cells within the infiltration remained largely unchanged, we next looked at the cellular composition. B cell (B220+) and T cell (CD3+) counts and organization did not differ among groups (B220: vs. IgG: p=0.080; CD3: vs. IgG: p>0.99) (**Figure 6A**), suggesting that the overall structure of the choroid plexus lymphoid infiltrate was not modified with treatment.

One of the important functions of TLS is the ability to produce antibodies localized to the site of inflammation (11). Cells co-stained with SDC1 and IgG indicate plasma cells producing antibodies. While plasma cells were present in the infiltrates, similar numbers were present across the groups (vs. IgG: p=0.64; **Figure 6B**). Lastly, we looked at cells that stained positive for activation-induced cytidine deaminase (AID), which is instrumental in B cells undergoing somatic hypermutation and class switching recombination. Positive staining for AID would indicate the presence of cells potentially making site-specific antibodies targeting the brain. Interestingly, both antibody groups, anti-CXCL13 treated and IgG control, had significantly fewer AID+ cells than the PBS group (**p=0.0041 and **p=0.0082, respectively), although they were not different from each other (p=0.83; **Figure 6C**).

### 3.5 Anti-CXCL13 Treatment Modifies Cortical and Hippocampal Gene Expression

Anti-CXCL13 treated mice showed significant improvement in the neurobehavioral abnormalities, despite no substantial changes in the histology or cell composition of the choroid plexus infiltration. We then explored potential changes on the molecular level. CXCL13 is largely known for lymphocyte recruitment and retention but is also linked to additional key intracellular signaling pathways, including ERK/MAPK and PI3K/Akt (31). A PCR array containing a set of genes important in neuroinflammation was used to assess gene expression in the cortex and hippocampus, including genes relevant to apoptosis, cellular stress, T cell activation and signaling, and inflammatory response pathways.

**Figure 7:**
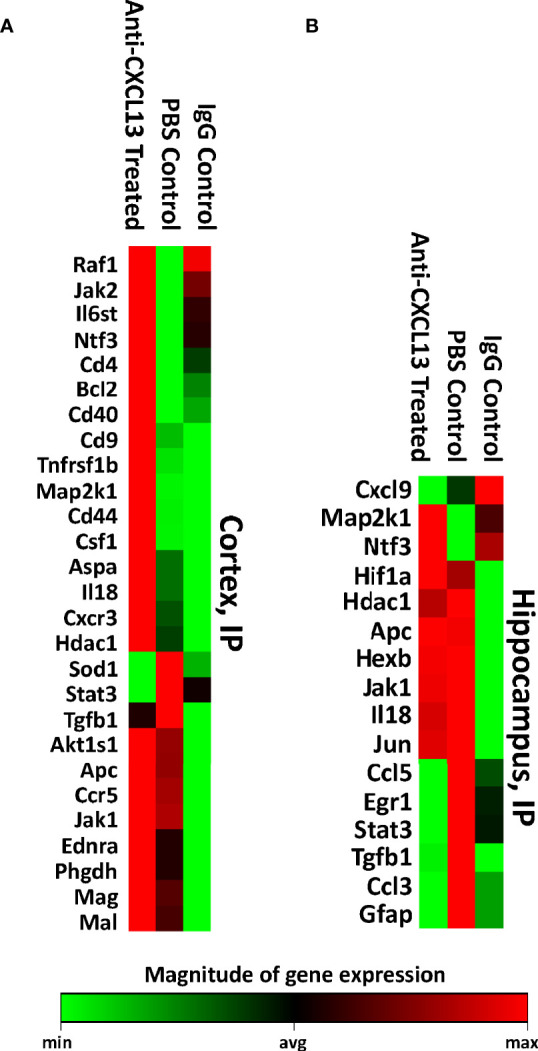
RNA expression levels of the cortex and hippocampus. Using the Qiagen RT^2^ Profiler PCR array, the **(A)** cortex and **(B)** hippocampus were measured for RNA expression levels of various genes related to neuronal apoptosis, inflammatory response, and other processes that may have contributed to the behavioral changes seen between the groups. All genes that were significantly different against the anti-CXCL13 treated group (p<0.05) are shown. Green indicates lower expression relative to the treatment group while red represents higher expression. PBS control: n=6, Anti-CXCL13 treated: n=6, IgG control: n=6.

Visual representations of genes that were significantly different between the anti-CXCL13 treated and IgG control group are presented in **Figures 7A, B**, and **Supplementary Figure 2**. While no single gene was consistently altered across groups and between tissues, specific genes that were decreased in the treated group included *SOD1* and *STAT3* (cortex) and *CXCL9* and *STAT3* (hippocampus), while *MAP2K1*, *HDAC1*, and *JAK1* were increased in the hippocampus. These and other genes altered with treatment included those with multiple functions. The expression array study indicates that with anti-CXCL13 treatment there was a significant change in the production of mediators that likely influenced neuroinflammation and other cellular processes.

### 3.6 Delivering the Treatment ICV Demonstrated Similar Results to IP Delivery

**Figure 8:**
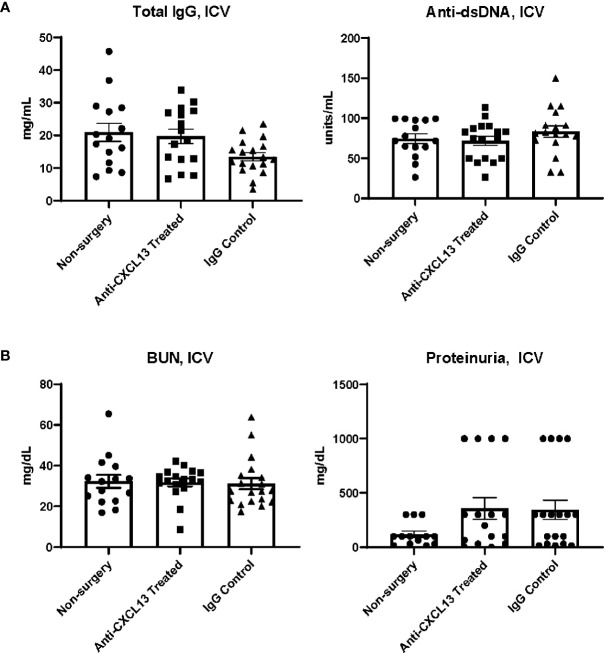
Systemic and renal disease indicators after ICV treatment delivery. With treatment delivered directly into the central nervous system, the **(A)** systemic and **(B)** renal disease indicators overall showed no improvement with anti-CXCL13 treatment. Total IgG: Anti-CXCL13 Treated: 19.72 ± 2.20 mg/mL and IgG Control: 13.47 ± 1.24 mg/mL, p=0.092; anti-dsDNA: Anti-CXCL13 Treated: 71.76 ± 5.75 units/mL and IgG Control: 83.27 ± 7.12 units/mL, p=0.40; BUN: Anti-CXCL13 Treated: 31.73 ± 1.94 mg/dL and IgG Control: 31.12 ± 2.78 mg/dL, p=0.71; Proteinuria: Anti-CXCL13 Treated: 357.80 ± 99.53 mg/dl and IgG Control: 344.70 ± 89.18 mg/dl, p>0.99. Non-surgery: n=15, Anti-CXCL13 treated: n=16, IgG control: n=17. Anti-dsDNA, anti-double stranded DNA; BUN, blood urea nitrogen.

The relative contribution of systemic disease to neuropsychiatric presentations in NPSLE is unclear since at times it is difficult to decide whether the brain manifestations are truly primary CNS disease or only secondary to systemic (extra-cranial) inflammation. Therefore, in addition to delivering the treatment IP, in a separate cohort in this study, we delivered the treatment intracerebroventricularly (ICV) to better discriminate between these possibilities. Delivering anti-CXCL13 directly into the cerebrospinal fluid facilitated the restriction of the treatment to the CNS (primarily) without affecting systemic inflammation, allowing any behavioral differences among groups to be fully explained by local changes in the brain. Cannulas with osmotic pumps were surgically implanted into two groups of female MRL/*lpr* mice at 15 weeks of age. One group received the anti-CXCL13 antibody while the other received the IgG isotype control antibody. Treatment was delivered continuously for two weeks, with all behavioral assessments given the week after, during the third week. All samples (serum, urine, tissue) were collected at the end of the third week. We saw no differences in the systemic disease parameters (Total IgG: vs. IgG: p=0.092; anti-dsDNA: vs. IgG: p=0.40; **Figure 8A**) or in renal function indicators (BUN: vs. IgG: p=0.71; proteinuria: vs. IgG: p>0.99; **Figure 8B**).

**Figure 9:**
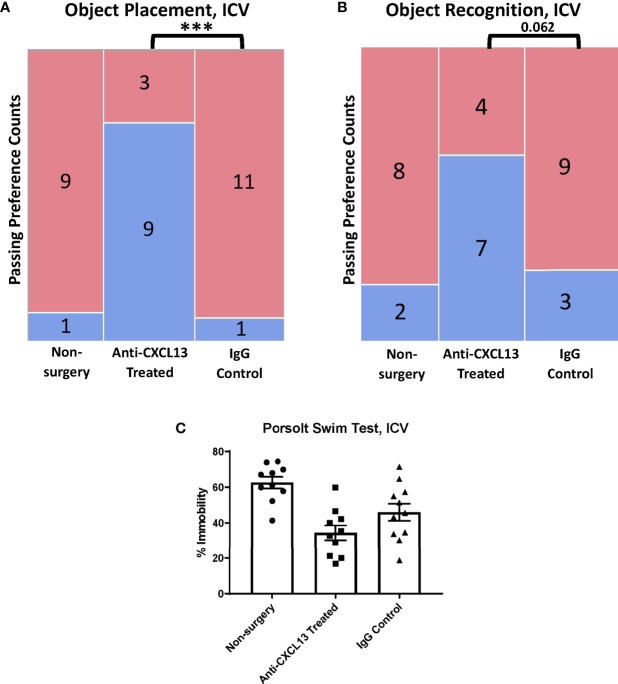
Behavioral assessment of the ICV cohort. **(A, B)** The anti-CXCL13 treated group had more mice show a preference (blue) over no preference (red) for the novel placement or object than the IgG control groups in both memory assessment tasks. OP task: Non-surgery: n=10, Anti-CXCL13 treated: n=12, IgG control: n=12; ***p=0.0009. OR task: Non-surgery: n=10, Anti-CXCL13 treated: n=11, IgG control: n=12; p=0.062. One mouse was excluded from the treated group in the OR task results due to an inadequate exploration time during the training trial. **(C)** Both antibody groups had significantly lower depression-like behavior than the non-surgery group. Anti-CXCL13 Treated: 34.33 ± 4.23% and IgG Control: 45.94 ± 4.79%, p=0.14. PBS control: n=10, Anti-CXCL13 treated: n=10, IgG control: n=11. One mouse in each of the treated and IgG groups was excluded after inability to float during the test. OP, object placement; OR, object recognition. ***p ≤ 0.001.

In the behavioral analysis, the ICV cohort showed significantly more mice pass in the treated group (75%) compared to the IgG control group (9.1%, ***p=0.0009) for the OP task (**Figure 9A**; **Supplementary Figure 1B**) while the OR task showed a similar trend towards significance (vs. IgG: p=0.062; **Figure 9B**; **Supplementary Figure 1B**). The Porsolt swim test results were not significant with ICV delivery (vs. the IgG: p=0.14; **Figure 9C**). These results point to significant attenuation in memory impairment with blocking CXCL13, independent of any differences in systemic disease.

**Figure 10:**
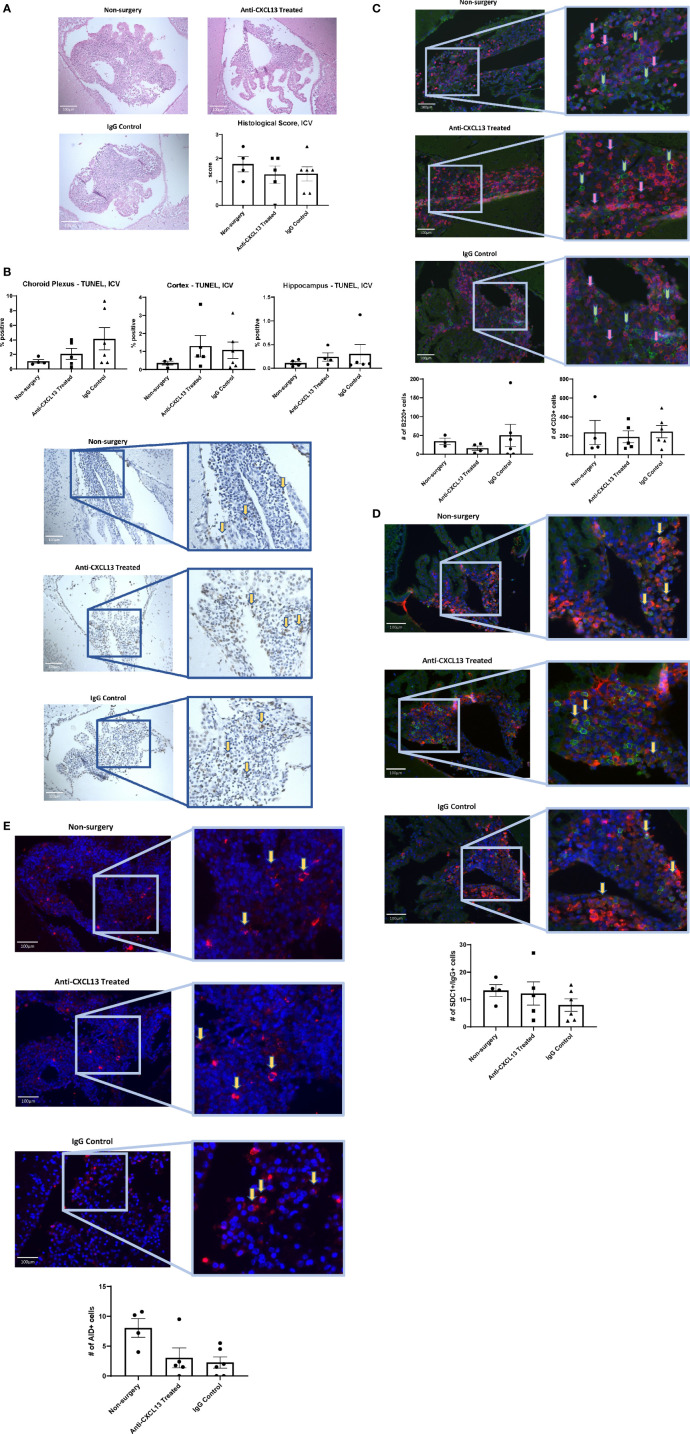
Choroid plexus infiltration characterization of the ICV cohort. The infiltration of the choroid plexus showed similar characteristics in the ICV cohort as it did in the IP cohort. There were no significant differences in **(A)** histology (Anti-CXCL13 Treated: 1.30 ± 0.37 and IgG Control: 1.33 ± 0.31, p=0.99) or **(B)** the percentage of apoptotic cells across the three tissues (choroid plexus, cortex, and hippocampus). (Choroid Plexus: Anti-CXCL13 Treated: 2.06 ± 0.76% and IgG Control: 4.13 ± 1.54%, p=0.42; Cortex: Anti-CXCL13 Treated: 1.30 ± 0.60% and IgG Control: 1.07 ± 0.47%, p=0.94; Hippocampus: Anti-CXCL13 Treated: 0.24± 0.089% and IgG Control: 0.30 ± 0.21%, p>0.99) The composition of the infiltration was also similar across the groups, as indicated by **(C)** B cell (B220+(green); Anti-CXCL13 Treated: 15.86 ± 6.06 cells and IgG Control: 50.00 ± 29.45 cells, p>0.99) and T cell (CD3+(magenta); Anti-CXCL13 Treated: 188.30 ± 62.15 cells and IgG Control: 244.30 ± 64.93 cells, p=0.87) clusters, **(D)** plasma cells (SDC1+(green)/IgG+(magenta); Anti-CXCL13 Treated: 12.19 ± 4.24 cells and IgG Control: 7.96 ± 2.29 cells, p=0.58), or **(E)** B cells undergoing antibody diversification (AID+(magenta); Anti-CXCL13 Treated: 2.02 ± 1.67 cells and IgG Control: 2.25 ± 0.94 cells, p>0.99). Histology of H&E stained slides was blindly scored by a veterinary pathologist. All images were taken at 20x magnification. Arrows point to several representative positively stained cells for illustration. The B220+ and CD3+ images have green and pink arrows to represent the two cell types, respectively. The images are representatives of each group, and quantification was calculated as averages of 3-4 images per mouse from a representative subset of the cohort. Non-surgery: n=4, Anti-CXCL13 treated: n=5, IgG control: 6. SDC1, syndecan-1; AID, activation-induced cytidine deaminase; TUNEL, terminal deoxynucleotidyl transferase dUTP nick end labeling; H&E, hematoxylin & eosin.

Histology and staining of the choroid plexus infiltration in ICV-treated mice were also similar to the IP cohort. H&E staining showed similar infiltration between groups (vs. IgG: p=0.99; **Figure 10A**), as were the apoptotic cell counts in the choroid plexus (vs. IgG: p=0.42; **Figure 10B**). As for cellular composition, the groups did not demonstrate significant differences in the B or T cell counts (B220: vs. IgG: p>0.99; CD3: vs. IgG: p=0.99; **Figure 10C**). Analysis of the plasma cell stain did not show differences between the anti-CXCL13 treated group and IgG control group (p=0.58) (**Figure 10D**). Lastly, AID+ cell counts did not reach significance in mice treated with anti-CXCL13 delivered ICV (vs. IgG: p>0.99; **Figure 10E**).

**Figure 11:**
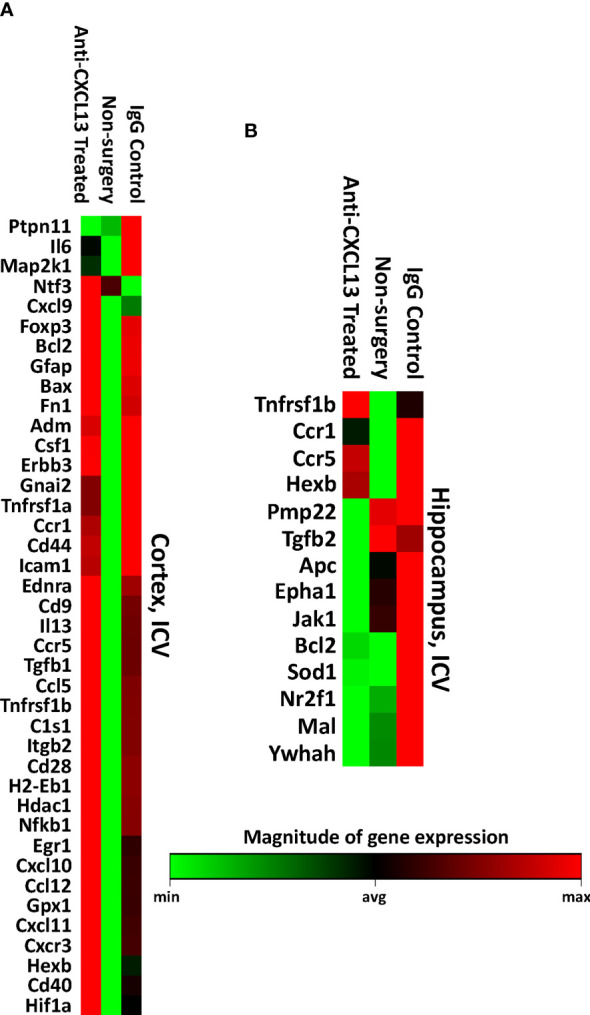
Brain gene expression levels of the ICV cohort. The **(A)** cortex and **(B)** hippocampus of a subset of the ICV cohort were measured for RNA expression levels using the same array as the IP cohort. All genes that were significantly different against the anti-CXCL13 treated group (p<0.05) are shown. The cortex has a number of upregulated genes in the two antibody groups compared to the non-surgery, most likely a result of the cannula implant surgery. Green indicates lower expression relative to the treatment group while red represents higher expression. PBS control: n=4, Anti-CXCL13 treated: n=5, IgG control: n=6.

RNA expression analysis of the cortex and hippocampus again found that anti-CXCL13 treatment induced changes in genes instrumental in processes such as inflammation and neuronal damage (**Figures 11A, B**; **Supplementary Figure 3**). The cortex of mice from the ICV cohort had a large number of genes significantly upregulated both in the anti-CXCL13 and IgG control groups compared to the baseline non-surgery group (**Figure 11A**; **Supplementary Figure 3A**). This latter finding was most likely due to damage done to the cortex from the implant surgery rather than an effect of delivering antibodies into the CNS. Nonetheless, *PTPN11* and *IL-6* were notably decreased in the cortex of anti-CXCL13 treated mice. In the hippocampus, which is removed from the area of surgery, anti-CXCL13 treated mice showed significant decreases in *EPHA1*, *JAK1*, and *BCL2* gene expression as compared to the IgG control group (**Figure 11B**; **Supplementary Figures 3A, C**).

## 4 Discussion

Currently, the treatment of NPSLE is not standardized as there is little evidence that any single medication or combination therapy works for all cases of NPSLE (32). Focusing specifically on the inflammatory presentation of NPSLE, current treatment recommendations differ based on disease severity; however, they still only have limited to moderate evidence of benefit or commonly lead to post-treatment relapse (4, 31). This unmet need in the treatment of NPSLE emphasizes the importance of studies that explore novel aspects of pathogenesis and investigate new therapeutic modalities.

TLS can form in a variety of contexts in autoimmune diseases, and accumulating evidence suggests that TLS develop in cases of SLE and may greatly impact disease progression (33). Evidence for TLS formation has been found in the skin, vasculature, and kidneys in SLE animal models, as well as in the kidneys of lupus patients (34–37). Chang et al. reported that half of the renal biopsies from LN patients had well-organized infiltrates in the tubulointerstitial space, demonstrating germinal center formation, as well as B cell clonal expansion and somatic hypermutation, suggesting antibody production (37). The kidney TLS were strongly associated with local immune complexes found in the tubular basement membrane, suggesting that TLS contributed to disease progression. Multiple sclerosis (MS) is a specific case in which TLS have been found in a neuroimmunological autoimmune disease, with antibodies proposed to recognize myelin and other neuronal components (11). Well-organized lymphocyte infiltrates in MS correlate with severe inflammation, as evident by increased meningeal and perivascular inflammation with B cells, plasma cells, and T cells found in the sulci of the frontal, temporal, and parietal lobes (38). Gatumu et al. demonstrated that blocking LTβR, an important cell recruitment factor in TLS formation, partially restored function of the salivary glands in NOD mice, a model for Sjogren’s syndrome, by reducing the size and number of follicles that formed and inhibiting CCL19 expression (39). Finally, we previously identified TLS in the brains of MRL/*lpr* mice, and previous studies reported the presence of CD45+ cells and immune complex deposition in the brains of NPSLE patients (6, 7, 40). These data collectively highlighted the potential importance of TLS in SLE and other autoimmune conditions (including those affecting the CNS) and suggested that interfering with TLS formation can be beneficial, which encouraged us to investigate the contribution of TLS to NPSLE pathogenesis and progression.

CXCL13 is a key component in lymphoid organization, including in TLS formation. Several studies show that mice deficient in CXCL13 or its receptor, CXCR5 (expressed on follicular dendritic cells and follicular T helper cells, respectively), lack peripheral lymph nodes and have reduced B-cell homing to follicles in existing lymph nodes and the spleen (12, 13, 41). Moreover, CXCL13 alone has been shown to be sufficient to initiate the formation of lymphoid structures in peripheral organs. Luther et al. reported that overexpression of CXCL13 under a pancreatic insulin promoter in diabetic rats induced pancreatic TLS formation (14). Additionally, local expression of CXCL13 positively correlates with TLS size and complexity (9), further implicating not just the importance of CXCL13 in TLS formation but also its influence on lymphoid tissue development.

Previous studies have looked at the association of CXCL13 and SLE specifically, reporting a positive correlation between CXCL13 and disease activity scores (15, 16, 42). Interestingly, separate from its chemotactic function, a direct role for CXCL13 in the pathogenesis of LN was suggested by Worthmann et al., who found that exposing cultured podocytes to CXCL13 increased secretion of CXCL12, MIF, ICAM-1, and VCAM-1 (43). Looking at CSF of patients with neurological diseases, CXCL13 was the cytokine most closely linked to B cell recruitment to the CNS and intrathecal Ig synthesis (44). The latter study emphasizes the importance of CXCL13 in the context of the CNS and the recruitment of B cells that may be contributing to disease.

In this study, we look at the effect of neutralizing CXCL13 in the context of NPSLE. Specifically, we assessed how treatment altered brain disease manifestations when delivered systemically or intracerebroventricularly. Treated mice showed less memory impairment and depression-like behavior, suggesting that CXCL13 contributes to the development or maintenance of these behavioral deficits and may likely play an important role in NPSLE pathogenesis. ICV administration of anti-CXCL13, which restricted the treatment to the CNS, allowed us to uniquely determine that the observed attenuation of the behavioral manifestations was due to changes in the brain disease progression and not simply secondary to any undetected changes in systemic disease.

The histological scores and immunofluorescence staining with cell counts indicated little change in terms of cellular infiltration extent and its overall composition, despite the expectation that neutralizing CXCL13 would either prevent recruitment of lymphocytes to the site or disrupt retention of the cells. The immune system, however, has proven redundancies, and it is likely that another similar chemokine, such as CCL20, could have recruited the cells in the absence of CXCL13. Cellularly, the one parameter that was different was the number of AID-positive cells between the two antibody groups and the control group not receiving active therapy. B cells positive for AID indicate germinal center development and increase of antibody diversity (45), suggesting the infiltrates in the antibody groups had less germinal center activity. As intravenous immunoglobulin (IVIg) is one of the established treatments for lupus (46), it could be that delivering any antibody alone, regardless of specificity, may decrease antibody diversification.

The PCR array assessed for RNA expression levels of genes involved in processes such as neuronal apoptosis, inflammatory response, and cell adhesion. CCL3 (or MIP-1α) is a chemokine secreted by macrophages that was significantly down-regulated in the hippocampus of the IP cohort. A previous study by Marciniak et al. demonstrated that injecting excess CCL3 into the brain significantly impaired performance in a Y-maze test (47). This study suggests that CCL3 is relevant to memory-based tasks and that decreased CCL3 in the treated group could have contributed to the improvement we saw in the OP task. CCL5 (or RANTES), a molecule found to be upregulated in CSF levels of NPSLE patients compare to non-NPSLE patients (48), was also down-regulated in the hippocampus of mice treated IP. In cases of HIV infection, CCL5 correlated with increased viral load and was one of the strongest predictors of neurocognitive impairment based on a global deficit score (49). Levels of CCL5 are elevated in the serum of patients with depression (50), suggesting, along with the connection with cognitive impairment and NPSLE, that the decrease of CCL5 in the treatment group may have contributed to the improvement in both behavioral indicators we tested for in this study.

A number of JAK/STAT pathway genes were altered with anti-CXCL13 treatment as well, with the most notable being STAT3, which was decreased in both cortex and hippocampus of the IP cohort. STAT3 is a key transcription factor in the development of Th17 cells, which have been hypothesized to be potential inducers of TLS formation, and also are involved in pathways regulating cell proliferation, differentiation, and inflammation (51, 52). Previous studies in MRL/*lpr* mice inhibiting STAT3 saw decreased anti-dsDNA antibodies and nephritis, as well as decreased renal tubulointerstitial lesions (53–55). Another study in rats following chronic mild stress correlated increased levels of STAT3 with increased immobility in the Porsolt swim test, implying STAT3’s potential role in affective deficits (56). Slight-Webb et al. suggested that the reduction of phosphorylated STAT3 is the primary mechanism responsible for disease suppression in lupus for mycophenolate mofetil, a current therapy for NPSLE (57). Surprisingly, all tissues (disregarding the cortex of the ICV cohort) showed up-regulation of *JAK1* or *JAK2*. Multiple studies showed that inhibiting Jak1 or Jak2 attenuated lupus-like disease in multiple mouse models (53), yet we saw an increase in the brains of the MRL/*lpr* strain. While the exact mechanistic changes are important to investigate and should be addressed in future studies, it is likely that unexpected changes in gene expression could have been the result of compensation by the immune system as the CXCL13 pathway was disrupted.

Another potential explanation of the neurobehavioral changes we saw in the treatment mice is that CXCL13 may be acting directly on brain cells (rather than mainly contributing to the TLS). CXCL13 is upregulated in the CNS in neuroinflammation by perivascular mononuclear cells and endogenous microglial in active MS plaques as well as by stromal cells in meningeal B cell aggregates (58). Additionally, animal studies have also not only demonstrated that CXCL13 may be produced by microglia with neuroinflammation but also that CXCR5 signaling in the brain can then lead to astrocyte activation (59) as well as affect the hippocampus to induce cognitive dysfunction (60). Furthermore, we previously described significantly increased CXCL13 and CXCR5 in the choroid plexus of MRL/*lpr* mice (8) and, in preliminary single-cell RNA-sequencing studies, determined that the cellular sources of these molecules are macrophages/microglia and stromal fibroblasts (for CXCL13) and B cells and double-negative T cells (for CXCR5) (data not shown). Choroid plexus tissue was however not obtained in our current study. While we did not find any significant change with treatment in CXCL13 or CXCR5 expression in the cortex and hippocampus (data not shown), this might be occurring in the choroid plexus, and/or might be detectable in the cortex and hippocampus by single cell rather than bulk analysis. In any case, the upregulated CXCL13/CXCR5 pathway in brain cells of MRL/*lpr* mice and the previous descriptions of enhanced brain CXCL13/CXCR5 expression in animal models provide a likely explanation for the beneficial effect of CXCL13 blockade in this study regardless of CNS leukocyte accumulation or TLS formation.

The current study demonstrated that treatment with an anti-CXCL13 neutralizing antibody attenuated behavioral deficits in lupus-prone mice and could be doing so on a molecular level by altering expression levels of certain inflammatory mediators or direct effect on resident brain cells. At least for the ICV cohort (e.g. **Supplementary Figure 3B**) we identified relatively few differentially expressed genes to fully explain the effects of treatment. However, the approach we chose was a selective array containing less than 100 genes, in particular pathways, dictated by the commercial product. A more comprehensive and unbiased characterization by bulk RNA sequencing may very well have disclosed many more gene differences. Nevertheless, the latter approach, while potentially helpful to further elucidate the basis for the therapeutic benefit of the antibody, was outside the scope of the present study and can be applied in future research to more definitively address the mechanism of benefit of anti-CXCL13 treatment in NPSLE. Moreover, we studied gene expression in the cortex and hippocampus, and as mentioned above the benefit we demonstrated was possibly derived in part from inhibition of the pathway in additional brain regions as well, e.g. the choroid plexus.

While our study suggests a promising new treatment approach to treat NPSLE, there are several limitations to consider. For one, we did not see systemic differences among the groups, even though previous studies have demonstrated effectiveness in blocking CXCL13 on autoimmunity. In experimental autoimmune encephalomyelitis (EAE), a MS murine model, blocking CXCL13 with an antibody or knocking it out entirely still allowed onset of disease to occur normally, but severity was attenuated over time, as shown by lower mean clinical scores and less activated microglia (61). In murine collagen-induced arthritis, a model of rheumatoid arthritis, blocking CXCL13 reduced disease severity and decreased synovial germinal center formation (62). Differences with our study may stem from how CXCL13 was blocked. The EAE study used a knockout mouse model (61); since complete depletion of CXCL13 has been shown to affect normal lymph node development (13), these differences in development may be why we did not see a similar improvement in systemic disease. The study utilizing collagen-induced arthritis mice neutralized CXCL13 using an antibody similarly to our study, but they do not specify the dosage or frequency of treatment (62), making it challenging to use for comparison. However, in a study by Finch et al. that specifically looked at lymphoid follicle development, CXCL13 neutralization in adult mice had no obvious effects on germinal centers, follicular dendritic cell networks, or Peyer’s patch structure (63). Their results are consistent with those of our study, suggesting that CXCL13 may have a more important role in lymphoid tissue formation than in its maintenance.

Interestingly, we also saw no differences in renal function in either of the two cohorts with the different delivery methods. Though improving renal dysfunction was not the focus of the present study, the results in the IP cohort differ from those reported by Wu et al. who saw significantly decreased proteinuria with CXCL13 blockade in MRL/*lpr* mice (17). These differences may be explained by differences in study design. Wu’s group, though delivering less per injection IP than we did in the present study (50 µg vs. 100 µg, respectively), treated the mice every day while we only treated three times a week. Moreover, the Wu study was also limited to 6 mice per group, whereas we studied groups of 13-21 mice; perhaps, with a larger sample size, the significant differences in systemic and renal disease initially reported would not have been confirmed. Our results also differed from a study done by Moreth et al. that looked at renal disease in MRL/*lpr* mice (64). They found that blocking the proteoglycan biglycan improves both systemic and renal parameters, which they attributed to declines in CXCL13 levels due to biglycan’s role in its regulation. However, biglycan also influences a number of other important inflammatory proteins, including TLR2 and TLR4, making it difficult to definitively attribute the beneficial effects of treatment in this study to any effects on the CXCL13 pathway.

One caveat of the current study was in the PCR array experiment; while still providing much information on the gene expression differences, a panel specific to NPSLE ideally would have been used, but since it is still unclear exactly what processes are involved in its pathogenesis, one could not be created accurately. Additionally, the ICV anti-CXCL13 treated and IgG control groups’ expression levels in the cortex had very similar profiles; this suggests that the surgery to implant the cannula did impact inflammation in the brain, as related genes were significantly lower in the non-surgery group. The hippocampus of the ICV cohort did not mirror this likely because the cannula does not cross the hippocampus at the coordinates we had chosen for its insertion.

A necessary consideration of the translatability of an anti-CXCL13 treatment is the delivery method. We used two different delivery methods, IP and ICV, in this study with lupus-prone mice. Both methods were effective in improving behavioral manifestations, but each poses individual concerns when considering administration in the clinical setting. Delivering the treatment systemically in humans will certainly have many off-target effects in secondary lymphoid organs. Since many SLE patients are already on immunosuppressants, blocking CXCL13 would serve to further dampen homeostatic immune responses. Delivering the treatment in a way that isolates it to the CNS would require an invasive approach, and infections are of major concern, such that ICV administration has little translational application at this time. Nevertheless, our study is one of the few that demonstrate that a systemically administered antibody can improve neuropsychiatric manifestations in a lupus-prone animal model.

Since we saw less than a full reversal of the NPSLE phenotype with CXCL13 inhibition, for future studies it may be beneficial to explore different dosages and/or frequency of delivery and to also test the same treatment in other lupus-prone mouse models with neuropsychiatric presentations to see if the treatment is equally advantageous. Additionally, some evidence suggests many lupus therapies would be effective concomitantly and should be studied in combination rather than one therapy at a time. A study by Sharma et al. demonstrated the effectiveness of neutralizing CXCL13 in conjunction with the BAFF receptor in a Sjogren model, preventing loss of salivary flow while reducing IgM levels (65). This may be the best approach with consideration of potential redundancies in the immune system, as discussed above. Especially if the TLS are an important contributing factor to the neuropsychiatric manifestations, targeting them by inhibiting certain components, such as CXCL13, or blocking certain functions, is worth pursuing further to develop more effective therapies for lupus and NPSLE.

## Data Availability Statement

The raw data supporting the conclusions of this article will be made available upon request from qualified individuals, without undue reservation.

## Ethics Statement

Animal studies were reviewed and approved by the Institutional Animal Care and Use Committee at Albert Einstein College of Medicine.

## Author Contributions

AS and CP conceived of the presented idea. MH designed the study with input from AS and CP. MH performed and analyzed all experiments. CP was in charge of overall direction and planning. MH and CP wrote the manuscript in consultation with all authors. All authors contributed to the article and approved the submitted version.

## Funding

The authors disclosed receipt of the following financial support for the research, authorship and/or publication of this article: NIH Training Program in Cellular and Molecular Biology and Genetics, T32 GM007491 (to MH).

## Conflict of Interest

The authors declare that the research was conducted in the absence of any commercial or financial relationships that could be construed as a potential conflict of interest.

## Publisher’s Note

All claims expressed in this article are solely those of the authors and do not necessarily represent those of their affiliated organizations, or those of the publisher, the editors and the reviewers. Any product that may be evaluated in this article, or claim that may be made by its manufacturer, is not guaranteed or endorsed by the publisher.

## Supplementary Material

Supplementary Figure 1 Object placement and object recognition task results of **(A)** the IP cohort and **(B)** the ICV cohort as represented by percent preference for the novel placement/object for individual mice. OP task, IP: PBS control: n=13, Anti-CXCL13 treated: n=19, IgG control: n=19; Anti-CXCL13 Treated: 58.63 ± 2.98% and IgG Control: 47.17 ± 4.75%; p=0.074. OR task, IP: PBS control: n=13, Anti-CXCL13 treated: n=19, IgG control: n=18; Anti-CXCL13 Treated: 56.56 ± 4.06% and IgG Control: 44.71 ± 3.80%; p=0.11. OP task, ICV: Non-surgery: n=10, Anti-CXCL13 treated: n=12, IgG control: n=12; Anti-CXCL13 Treated: 57.99 ± 3.55% and IgG Control: 37.33 ± 4.63%; p=0.0015. OR task, ICV: Non-surgery: n=10, Anti-CXCL13 treated: n=11, IgG control: n=12; Anti-CXCL13 Treated: 54.21 ± 5.86% and IgG Control: 39.13 ± 5.80%; p=0.13. The red dashed line represents the cut-off for preference versus no preference at 55%.

Supplementary Figure 2
**(A)** RNA expression levels in the cortex and hippocampus for individual mice following systemic antibody administration are shown by heat map. All genes that were significantly different against the anti-CXCL13 treated group (p<0.05) are shown. Green indicates lower expression and red indicates higher expression relative to the treatment group. Venn diagrams show the differential genes between the anti-CXCL13 treated and IgG control groups, with enrichment analysis describing their biological processes, for **(B)** the cortex and **(C)** the hippocampus. The number of genes not significantly different between the groups is presented in the intersection of the two Venn diagram circles. PBS control: n=6, Anti-CXCL13 treated: n=6, IgG control: n=6.

Supplementary Figure 3
**(A)** RNA expression levels in the cortex and hippocampus for individual mice following ICV antibody administration are shown by heat map. All genes that were significantly different against the anti-CXCL13 treated group (p<0.05) are shown. Green indicates lower expression and red indicates higher expression relative to the treatment group. Differential genes between the anti-CXCL13 and IgG control groups are shown in the Venn diagrams for **(B)** the cortex and **(C)** the hippocampus. Enrichment analysis was done for the hippocampus but not the cortex as there were too few genes. The number of genes not significantly different between the groups is presented in the intersection of the two Venn diagram circles. PBS control: n=4, Anti-CXCL13 treated: n=5, IgG control: n=6.

## Abbreviations

NPSLE, neuropsychiatric lupus; SLE, systemic lupus erythematosus; TLS, tertiary lymphoid structures; ICV, intracerebroventricularly; OP, object placement; OR, object recognition; BUN, blood urea nitrogen; H&E, hematoxylin and eosin; SDC1, syndecan-1; AID, activation-induced cytidine deaminase; TUNEL, terminal deoxynucleotidyl transferase dUTP nick end labeling.
