# Supplementary figures and images for: CXCL13 Neutralization Attenuates Neuropsychiatric Manifestations in Lupus-Prone Mice
Source: Front Immunol. 2021 Nov 12;12:763065. doi: 10.3389/fimmu.2021.763065 (PMC8633419; doi:10.3389/fimmu.2021.763065)

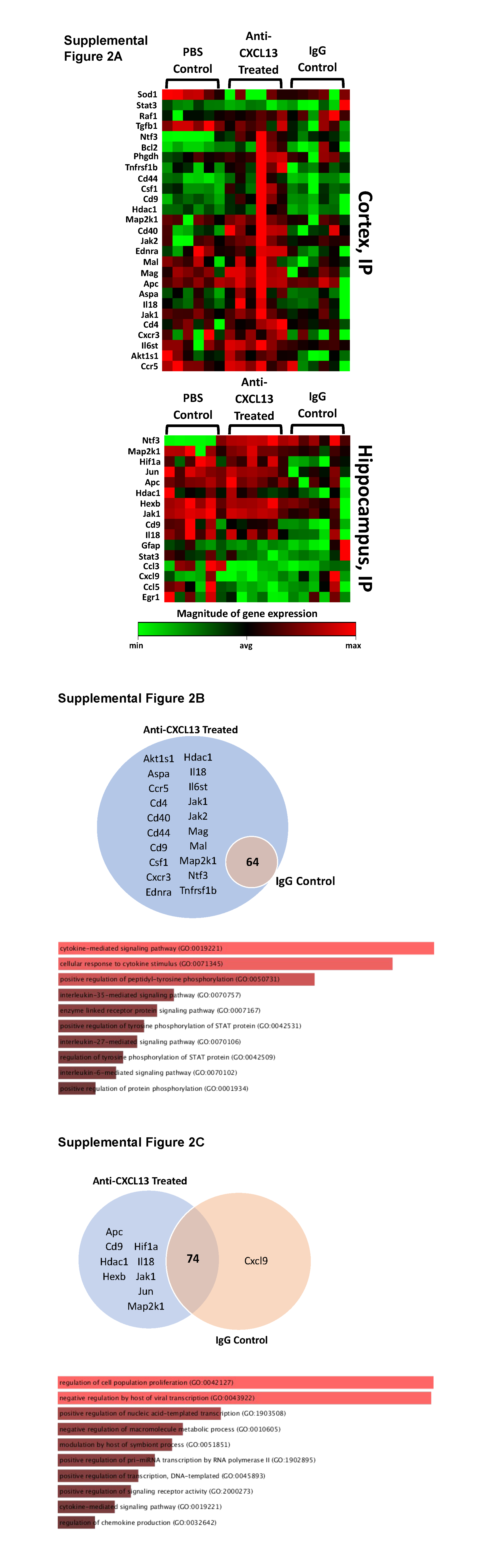

Supplement: Supplementary Figure 2 — (A) RNA expression levels in the cortex and hippocampus for individual mice following systemic antibody administration are shown by heat map. All genes that were significantly different against the anti-CXCL13 treated group (p<0.05) are shown. Green indicates lower expression and red indicates higher expression relative to the treatment group. Venn diagrams show the differential genes between the anti-CXCL13 treated and IgG control groups, with enrichment analysis describing their biological processes, for (B) the cortex and (C) the hippocampus. The number of genes not significantly different between the groups is presented in the intersection of the two Venn diagram circles. PBS control: n=6, Anti-CXCL13 treated: n=6, IgG control: n=6. [file Image_2.tif]

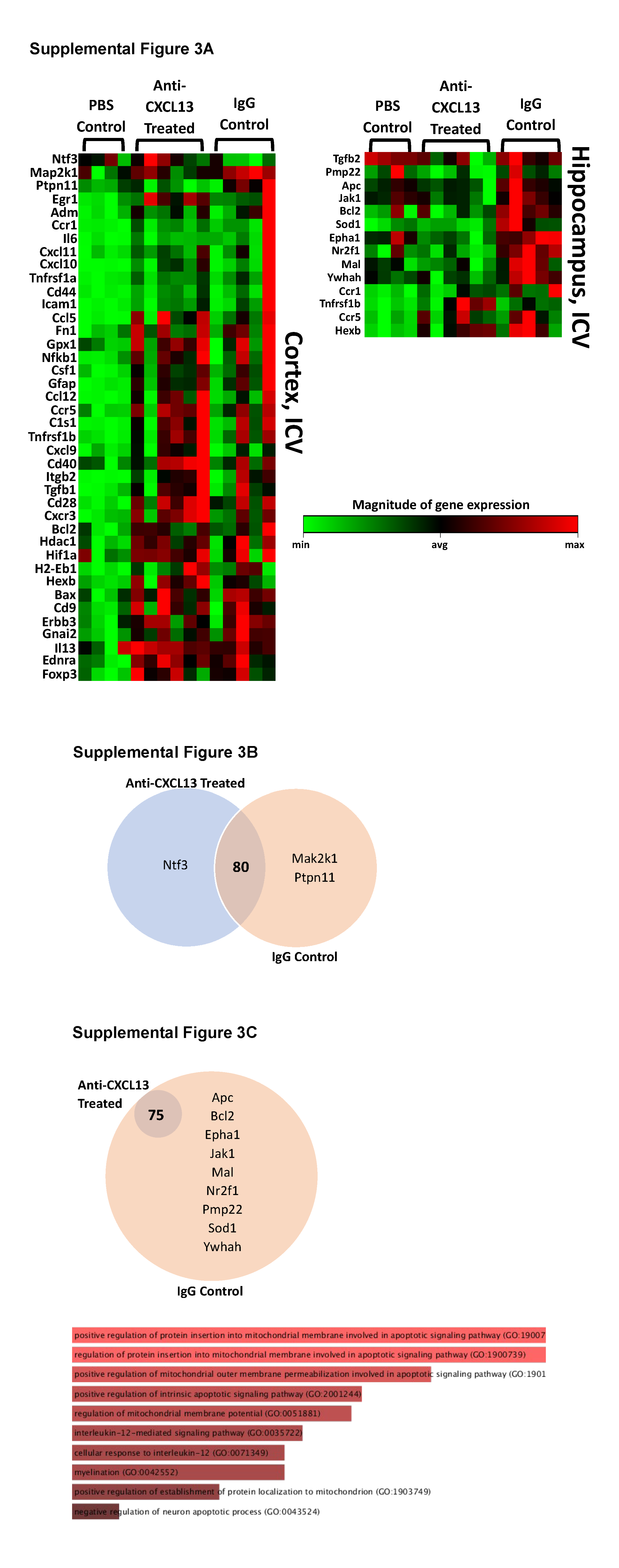

Supplement: Supplementary Figure 3 — (A) RNA expression levels in the cortex and hippocampus for individual mice following ICV antibody administration are shown by heat map. All genes that were significantly different against the anti-CXCL13 treated group (p<0.05) are shown. Green indicates lower expression and red indicates higher expression relative to the treatment group. Differential genes between the anti-CXCL13 and IgG control groups are shown in the Venn diagrams for (B) the cortex and (C) the hippocampus. Enrichment analysis was done for the hippocampus but not the cortex as there were too few genes. The number of genes not significantly different between the groups is presented in the intersection of the two Venn diagram circles. PBS control: n=4, Anti-CXCL13 treated: n=5, IgG control: n=6. [file Image_3.tif]
